# Supplementary material for: Complexome profiling of the Chlamydomonas psb28 mutant reveals TEF5 as an early PSII assembly factor
Source: Plant Cell. 2025 Mar 17;37(6):koaf055. doi: 10.1093/plcell/koaf055 (PMC12164590; doi:10.1093/plcell/koaf055)
Supplement: koaf055_Supplementary_Data [file koaf055_supplementary_data.zip › psb28_interactive.zip!/plots/intensity/qualityPlots/peptides/GAS31_Cre11.g468359.t1.1.html]

### Cre11.g468359.t1.1

Trivial name: GAS31  
SeqCoverage [median]: 3.80  
Mass: 71.56 kDa  
  
 

| Sequence | MissedCleavage | Mass [Da] | wtIntensity | muIntensity | Score |
| --- | --- | --- | --- | --- | --- |
| DAGGSYVDATVPGNR | 0 | 1477.68 | 379.02 | 800.21 | 138.99 |
| VYTYDGNELDVR | 0 | 1442.67 | 734.92 | 1367.39 | 80.76 |
|  |  |  | 1113.9 | 2167.6 |  |
